# P069: Drug-resistant acinetobacter ventilator-associated pneumonia: a time for desperate measures!

**DOI:** 10.1186/2047-2994-2-S1-P69

**Published:** 2013-06-20

**Authors:** HH Balkhy, A El-Saed, R Maghraby, HH Al-Dorzi, R Khan, AH Rishu, YM Arabi

**Affiliations:** 1 Department of Infection Prevention and Control, King Abdulaziz Medical City, Riyadh, Saudi Arabia; 2 Department of Pediatrics, King Abdulaziz Medical City, Riyadh, Saudi Arabia; 3 Intensive Care Department, King Abdulaziz Medical City, Riyadh, Saudi Arabia

## Introduction

There is a wide geographic and temporal variability of bacterial resistance among microbial causes of ventilator-associated pneumonia (VAP). The contribution of multi-drug resistant (MDR) pathogens to the VAP etiology in Saudi Arabia was never studied.

## Objectives

We sought to examine the extent of multiple-drug resistance among common microbial causes of VAP.

## Methods

We conducted a retrospective susceptibility study in the adult ICU of King Abdulaziz Medical City, Riyadh, Saudi Arabia. Susceptibility results of isolates from patients diagnosed with VAP between October 2004 and June 2009 were examined. The US National Healthcare Safety Network (NHSN) definition of MDR was adopted.

## Results

A total of 248 isolates including 9 different pathogens were included. *Acinetobacter* spp. was highly (70-90%) resistant to all tested antimicrobials including carbapenems (three- and four-class MDR prevalence were 86% and 78%, respectively). *Pseudomonas aeruginosa* was moderately (20-40%) resistant to all tested antimicrobials including antipseudomonal penicillins(three- and four-class MDR prevalence were 18% and 10%, respectively). With exception of ampicillin (fully resistant), *Klebsiella* spp. had low (0-14%) resistance to other tested antimicrobials with no detected MDR. *Staphylococcus aureus*was fully susceptible to vancomycin with 42% resistance to oxacillin. There were significant increasing trends of MDR *Acinetobacter*spp. but not *Pseudomonas aeruginosa* during the study.

## Conclusion

*Acinetobacter*in the current study was an increasingly resistant VAP-associated pathogen more than seen in many parts of the world. The current finding may impact local choice of initial empiric antibiotic and emphasize the need to improve currently implemented antimicrobial stewardship and environmental cleaning. Measures to reduce the burden of this organism from such sites may assist in reducing the burden of *Acinetobacter* as a human pathogen in healthcare settings.

## Disclosure of interest

None declared

